# Agreement between swept-source OCT and Scheimpflug biometers in ocular measurements

**DOI:** 10.1186/s12886-025-03916-0

**Published:** 2025-03-12

**Authors:** Cameron McLintock, James McKelvie, Hamed Niyazmand, Samir Uprety

**Affiliations:** 1 Department of Ophthalmology, Princess Alexandra hospital, Brisbane, QLD Australia; 2 Faculty of Medicine, University of Queensland, 199 Ipswich Road, Woolloongabba, Brisbane, QLD 4102 Australia; 3 Department of Ophthalmology, University of Auckland, Auckland, New Zealand; 4 Division of Optometry, School of Allied Health, Faculty of Health and Medical Sciences, The University of Western Australia, Crawley, WA Australia; 5 Vision for Life Institute, Brisbane, QLD Australia

**Keywords:** IOLMaster 700, Galilei G6, Agreement, Posterior keratometry, Total keratometry

## Abstract

**Background:**

This study evaluated the agreement of the ocular parameters obtained with the two optical biometers, the IOLMaster 700 and the Galilei G6 Lens Professional.

**Patients and methods:**

A comparative prospective study was conducted on 159 eyes of 91 adult patients using the IOLMaster 700 and Galilei G6 devices by a single examiner. Agreement between ocular biometric parameters: white-to-white (WTW) distance, keratometry (flat (K1) and steep (K2), mean (Km)) of anterior, posterior, and total corneal surfaces, central corneal thickness (CCT), anterior chamber depth (ACD), and axial length (AL) were assessed using Bland-Altman analysis. Keratometry measurements were further transformed into power vector components J0 and J45 for astigmatism analysis. Clinically significant differences were defined as deviations in biometric parameters translating to differences of 0.25 D or more in refractive outcomes.

**Results:**

Statistically and clinically significant difference was identified for ACD (mean difference: -0.15 mm), posterior corneal metrics: K1 (0.39 D), K2 (0.42 D), Km (0.41 D) and J0 (0.05 D) and total corneal metrics: K1 (0.95 D), K2 (0.91 D), Km (0.93 D) and J0 (0.13 D). No significant differences were found for J45 components of posterior and total K, WTW, CCT, and AL measurements.

**Conclusion:**

The difference in measurements of anterior chamber depth (ACD), posterior K, and total K metrics are clinically significant making the two devices are clinically significant and not interchangeable. These variation in metrics can impact the refractive outcomes of refractive and cataract surgery with toric IOLs.

## Background

The IOLMaster 700 (Carl Zeiss Meditec AG, Jena, Germany) is widely used swept-source optical coherence (SS-OCT) based optical biometry device [1] that has recently been updated to directly measure the posterior and total corneal powers (TK^®^) [2]. The Galilei G6 Lens Professional (Ziemer Ophthalmic Systems AG, Port, Switzerland) is another commonly used optical biometer that utilises the Placido disc-combined dual rotating Scheimpflug cameras, and an OCT based A-scan [3] to measure ocular biometrics including anterior and posterior corneal surfaces [4, 5].

Previous studies have shown that Galilei G6 demonstrates good agreement with existing biometers, such as Anterion (Heidelberg Engineering, Heidelberg, Germany) [4], Lenstar (Haag-Streit Koeniz, Switzerland) [6], IOLMaster 500 [7], and IOLMaster 700 in ocular biometrics [8, 9]. However, none of these studies have compared posterior and total corneal measurements. Incorporating these corneal metrics for intraocular lens (IOL) calculations has been demonstrated to improve reactive outcomes compared to conventional keratometry measurements [10]. Posterior corneal measurements are clinically relevant, as neglecting them may result in unexpected refractive outcomes, especially with toric IOL implantation [11]. Additionally, measuring posterior curvature improves the diagnostic accuracy of corneal ecstatic conditions such as keratoconus, and changes in these measurements aid in monitoring disease progression [12]. Therefore, this study aimed to evaluate the agreement between these two devices across ocular biometrics, including posterior keratometry, and total keratometry measurements.

## Materials and methods

The study was prospective and comparative in design. Data were obtained from patients attending the Princess Alexandra Hospital (Ophthalmology Department), Brisbane, Australia. All patients underwent a comprehensive ocular health assessment, including optic nerve and macula OCT scans prior to biometry measurements.

According to manufactures instructions, both the biometers were first calibrated at the start of the measurement each day. Prior to testing, patients were advised not to use any eye drops. A single examiner performed all the biometry measurements with IOLMaster 700 to scan both eyes, followed by the Galilei G6. Because of the built-in multiple measurement (least 3 to 5 repeat) that provides average results of the scans, we opted to use a single measurement for analysis. The quality of the measurements was ensured by adhering to quality-scoring metrics of each device. If the scans did not adhere the quality check of the system, we performed three additional attempts to obtain a quality scan. If the scan quality was still poor, the scan was not included in the study.

Exclusion criteria included patients with poor fixation and eyes with any ocular morbidities that were likely to alter the measurements, such as pterygium, corneal pathologies altering corneal dimensions such as scarring, keratoconus, corneal dystrophy, and retinal pathologies such as cystoid macular edema or posterior staphyloma.

Data from both eyes were included in the analysis.

Statistical analysis was performed using SPSS version 25 (IBM Corporation). Normality was checked using the Wilks-Shapiro test. Biometry parameters: white-to-white (WTW), central corneal thickness (CCT), keratometry (flat: K1, steep: K2, and mean: Km) for the anterior, posterior, and total cornea, anterior chamber depth (ACD), lens thickness (LT), and axial length (AL) were assessed. All keratometry data were transformed into power vectors (J0, J45) for astigmatic analysis [13].

Bland-Altman analysis was performed to evaluate the agreement between the two devices. The limits of agreement (LoA) were computed as the mean difference ± 1.96 standard deviations. Generalized Estimating Equations with Bonferroni correction for multiple comparisons were employed to compare if the device means differed significantly while controlling for inter-eye correlation. A p-value greater than 0.05 was considered statistically significant. A clinical significance criterion was set if the mean difference for a given metric between devices resulted in a refractive change of ≥ 0.25D.^14^ Intraclass Correlation Coefficient (ICC) was used to assess the reliability between measurements obtained from the IOLMaster 700 and Galilei G6. A two-way mixed-effects model, assuming absolute agreement was used to estimate the reliability coefficient for each variable. A post hoc analysis, computed using G*Power version 3.1 with a small effect size (0.3) and a Type I error rate set at 0.05, resulted in 96% power with a total sample size of *N* = 159.

## Results

A total of 182 eyes of 91 patients were enrolled in the study. Twenty-three (12%) eyes were excluded from the study due to retinal pathologies and measurements that did not adhere to the quality check of the system. Only 159 eyes were included in the analysis, with a male-to-female ratio of 0.82 and a mean participant age of 57 ± 18 years. Table 1 shows descriptive statistics (mean and standard deviation (SD)), results from Bland-Altman analysis and interclass correlation coefficients for each measurement between two devices.

**Table 1:** Agreement of biometry metrics between IOLMaster 700 and Galilei G6 Lens Professional. P-values derived from paired t-tests, Bonferroni correction is applied manually. Mean difference and limits of agreement are calculated based on the bland-Altman analysis. D = diopter, LoA = limit of agreement, ICC = intraclass coefficient of correlation

Variable	IOLmaster Mean ± SD	Galilei Mean ± SD	*P*-value	Mean difference	Lower LoA	Upper LoA	Mean Absolute difference	ICC
Anterior flat K (K1, D)	43.46 ± 1.65	43.39 ± 1.64	*p* > 0.05	0.06	-0.96	1.09	0.26	0.974
Anterior steep K (K2, D)	44.31 ± 1.63	44.23 ± 1.63	*p* > 0.05	0.08	-0.84	1.00	0.28	0.978
Anterior mean K (Km, D)	43.92 ± 1.55	43.85 ± 1.55	*p* > 0.05	0.07	-0.83	0.98	0.24	0.977
Anterior J0 (D)	-0.11 ± 0.43	-0.15 ± 0.40	*p* > 0.05	0.03	-0.32	0.40	0.13	0.947
Anterior J45 (D)	0.02 ± 0.24	0.00 ± 0.20	*p* > 0.05	0.02	-0.33	0.38	0.11	0.802
Posterior flat K (K1, D)	-5.71 ± 0.19	-6.11 ± 0.30	*p* < 0.001	0.39	0.00	0.78	0.41	0.472
Posterior steep K (K2, D)	-6.01 ± 0.26	-6.43 ± 0.44	*p* < 0.001	0.42	-0.20	1.04	0.42	0.548
Posterior mean K (Km, D)	-5.86 ± 0.21	-6.27 ± 0.36	*p* < 0.001	0.41	-0.05	0.87	0.41	0.520
Posterior J0 (D)	0.13 ± 0.06	0.08 ± 0.14	*p* = 0.04	0.05	-0.22	0.32	0.07	0.349
Posterior J45 (D)	0.01 ± 0.08	0.00 ± 0.11	*p* > 0.05	0.01	-0.17	0.21	0.06	0.652
Total flat K (K1, D)	43.83 ± 1.48	42.87 ± 1.41	*p* < 0.001	0.95	-0.01	1.92	0.95	0.872
Total steep K (K2, D)	44.65 ± 1.47	43.73 ± 1.43	*p* < 0.001	0.91	0.14	1.68	0.91	0.891
Total mean K (Km, D)	44.24 ± 1.44	43.30 ± 1.39	*p* < 0.001	0.93	0.18	1.69	0.93	0.884
Total J0 (D)	0.05 ± 0.43	-0.08 ± 0.45	*p* < 0.001	0.13	-0.28	0.56	0.19	0.914
Total J45 (D)	0.04 ± 0.23	0.00 ± 0.23	*p* > 0.05	0.04	-0.37	0.46	0.14	0.739
Lens Thickness (mm)	4.239 ± 0.615	4.196 ± 0.497	*p* > 0.05	0.042	-0.844	0.929	0.222	0.804
CCT (mm)	0.533 ± 0.034	0.534 ± 0.030	*p* > 0.05	-0.001	-0.025	0.024	0.008	0.960
ACD (mm)	3.384 ± 0.567	3.540 ± 0.560	*p* < 0.001	-0.155	-0.524	0.213	0.166	0.953
WTW (mm)	12.081 ± 0.425	12.084 ± 0.409	*p* > 0.05	-0.002	-0.490	0.484	0.149	0.903
Axial Length (mm)	24.016 ± 1.639	24.044 ± 1.67	*p* > 0.05	-0.027	-0.339	0.283	0.077	0.998

**Fig. 1:**
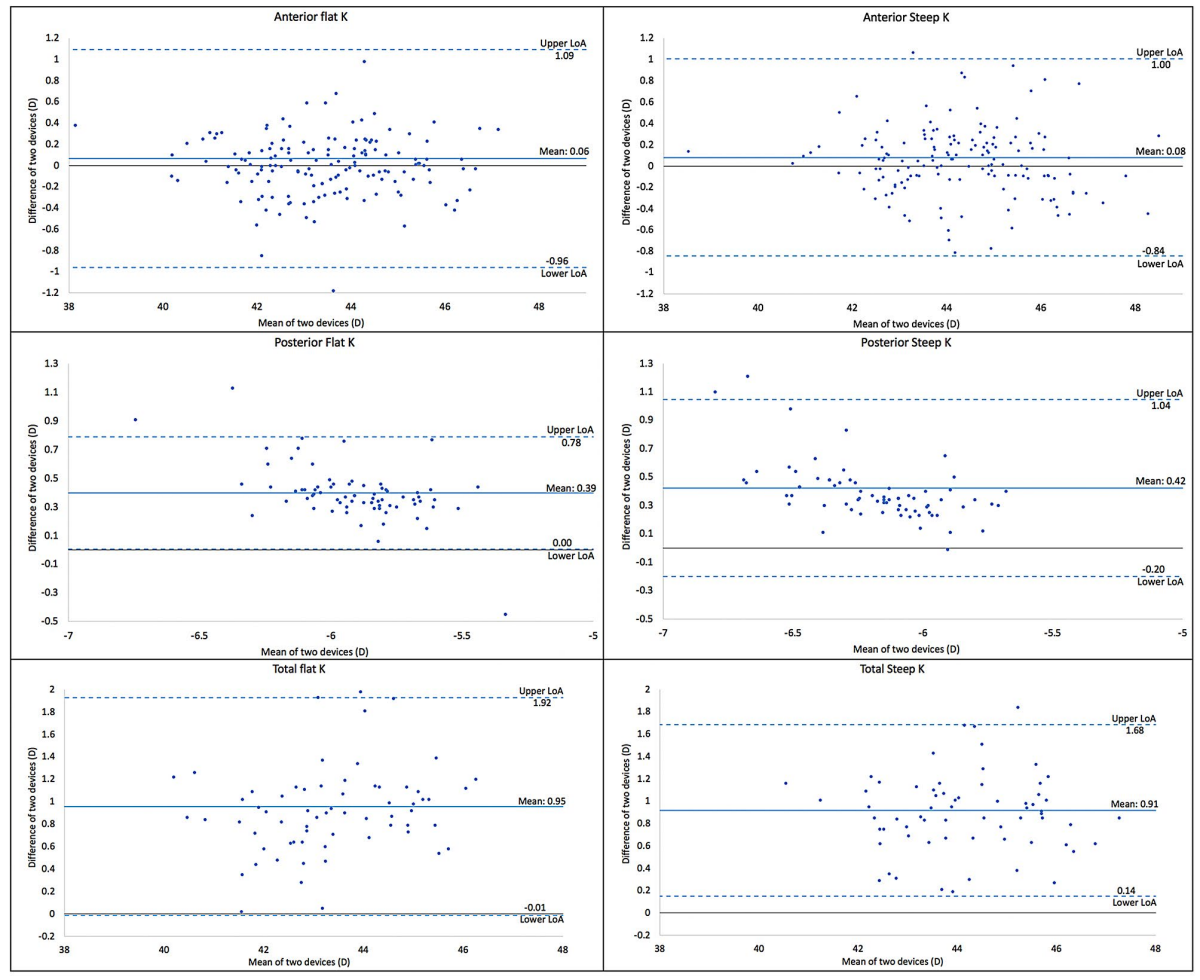
Bland-Altman plot for corneal metrics: Anterior (K1, K2), Posterior (K1, K2), Total (K1, K2)

**Fig. 2:**
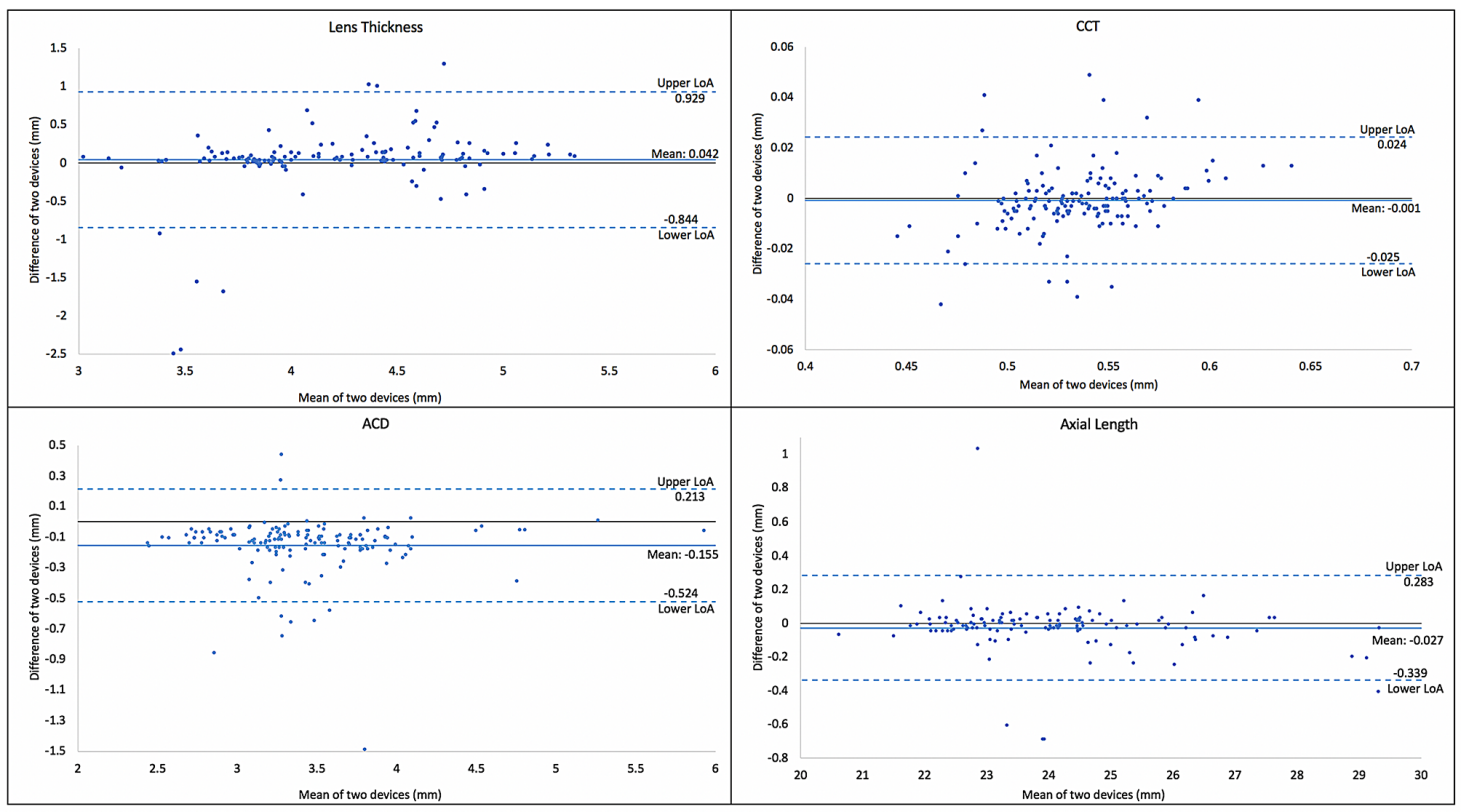
Bland-Altman plot for Lens thickness (LT), Anterior Chamber Depth (ACD), Central Corneal Thickness (CCT) and Axial Length (AL)

For anterior K measurements and their vector components, there was a comparable mean difference [K1: MD = 0.06 D; K2 = 0.08 D; Km = 0.07 D; J0 = 0.03 D, and J45 = 0.02 D] between the two devices (Table 1). The 95% limits of agreement ranged from K1: -0.96 D to 1.09 D; K2: -0.84 D to 1.0 D; Km: -0.83 D to 0.98 D; J0: -0.32 D to 0.40 D, and J45: -0.33 D to 0.38 D, indicating good agreement between the devices (Fig. 1, 2). The Intraclass Correlation Coefficient (ICC) calculated for all anterior K measurements and their vector components showed good reliability (> 0.8) between the devices.

Posterior K measurements and their vector components showed a significant difference (*p* < 0.05) in the mean difference between the two devices for K1 = 0.39 D; K2 = 0.42 D; Km = 0.41 D, and J0 = 0.05 D (Table 1). The 95% limits of agreement ranged from 0.00 D to 0.78 D for K1; -0.20 D to 1.04 D for K2; -0.05 D to 0.87 D for Km; and − 0.22 D to 0.32 D for J0, indicating considerable variability between the devices (Fig. 2). However, the J45 component showed comparable results (MD = 0.01 D) and good agreement (LoA = -0.22 D to 0.32 D) between the two devices. Poor reliability (ICC: 0.3 to 0.5) was observed for all posterior K measurements and the J0 vector component.

Similarly, total K measurements and their vector components showed a significant difference (*p* < 0.05) in the mean difference between the two devices for K1 = 0.95 D; K2 = 0.91 D; Km = 0.93 D, and J0 = 0.13 D (Table 1). The 95% limits of agreement ranged from − 0.01 D to 1.95 D for K1; 0.14 D to 1.68 D for K2; 0.18 D to 1.69 D for Km; and − 0.13 D to 0.28 D for J0, indicating considerable variability between the devices (Fig. 2). However, the J45 component showed comparable results (MD = 0.04 D) and good agreement (LoA = -0.37 D to 0.46 D) between the two devices. The Intraclass Correlation Coefficient (ICC) calculated for all total K measurements and their vector components showed good reliability (> 0.8) between the devices.

Between the two devices, there was a significant difference (*P* < 0.001) in ACD measurement, with a mean difference of -0.15 mm (LoA: -0.524 mm to 0.213 mm). However, for LT (MD = 0.042 mm; LoA = -0.844 mm to 0.929 mm), CCT (MD = -0.001 mm; LoA = -0.025 mm to 0.024 mm), WTW (MD = -0.002 mm; LoA = -0.490 mm to 0.484 mm), and AL (MD = -0.027 mm; LoA = -0.339 mm to 0.283 mm) there was no statistically significant difference between the two devices (Fig. 2). For the ACD, CCT, LT, WTW, and AL parameters, the Intraclass Correlation Coefficient (ICC) was > 0.8, indicating good device reliability (Table 1).

## Discussion

The cornea serves as principal refractive element of the human eye, and true measurement of its power is vital for accurate estimation of IOL power [14]. Precise measurement of anterior and posterior corneal surface is required to estimate the true total corneal power [15]. To authors knowledge, no other reports have compared posterior and total keratometry measurements between IOLMaster 700 and the Galilei G6. The difference in posterior and total corneal metrics between the two devices was statistically and clinically significant (> 0.25 D) [16]. The only posterior corneal metric for which no significant difference was found between devices was J45. This is explained by the fact that most eyes in this series had with-the-rule posterior corneal astigmatism, under which circumstances the J45 vector magnitude is small and any differences between devices are likely to be negligible. The difference in the corneal metrics found in this study may be due to the use of unique principle adopted by each device. While the Galilei derives the posterior corneal metrics from dual rotating Scheimpflug cameras [5], The IOLMaster 700 employs a combination of telecentric keratometry and SS-OCT-derived corneal thickness measurements to compute the posterior corneal surface [17]. Additionally, the IOLMaster derives the keratometry measurements from a 3.5 mm zone, while the Galilei derives these values from a zone with an inner radius of 1.0 mm and an outer radius of 4.0 mm [18]. This difference in zonal variations may also have contributed to the disagreement of posterior and total keratometry measurements found here, because the cornea is aspheric on both surfaces, and the curvature measurements alter as a function of zone of measurement [19] The authors infer that there is poor interchangeability between the two devices when comparing posterior and total keratometry metrics. The interchangeability of such metrics becomes particularly significant, when multiple devices are employed for estimating biometry measurements of the eye.

The comparison of anterior chamber depth (ACD) showed a significant difference (mean difference: -0.155 mm) between the two devices (Table 1). Anterior chamber depth measurement is crucial for precise intraocular lens power computation, as it determines the post-operative effective lens position. Depending on the axial length parameter, an ACD error of 0.25 mm can lead to a refractive outcome change ranging from 0.1 D to 0.55 D [20]. Our study found a mean ACD difference of 0.15 mm that could result in a refractive outcome difference of more than 0.2 D, particularly in shorter eyes. This underscores the clinical relevance of device specific ACD measurements and their potential impact on surgical outcomes. In contrast to our results, smaller mean differences in ACD between devices were reported by several studies with mean difference ranging from 0.05 to 0.07 mm [8, 9, 21, 22].

Anterior corneal metrics showed no significant difference between the devices. In contrast, Jung et al. [9] reported significant differences between devices for K1 (MD = 0.15D) and Km (MD = 0.11D), while Henriquez et al. [8] reported larger differences for anterior K1 (MD = -0.25D), K2 (MD = -0.13D), and Km (MD = 0.23D) compared to the present study.

No significant difference was found in CCT measurements between devices (MD = -0.001 mm), similar to Henriquez et al. (MD = -0.0015 mm) [8]. In contrast, Jung et al. [9] found a significant difference between devices (MD = -0.17 mm). Such discrepancies in CCT measurements may be significant in formulas such as Kane and Olsen [20], which incorporate CCT values to compute intraocular lens power. Translating the maximum mean difference in CCT reported by studies could produce clinically significant refractive change of approximately 0.25 D [23]. Therefore, the interchangeability of CCT measurements between devices should be used cautiously.

The agreement of LT, WTW, and AL between devices was similar to that reported in published studies [8, 9]. The difference in axial length between IOLMaster 700 and Galilei G6 did not produce clinically significant changes in refractive outcomes, considering that a 1 mm change can produce a 2.5 D change in refraction [24]. This suggests that the interchangeability of these parameters between the devices can be considered.

As our study found notable variations in posterior K, total K, and ACD between the two devices, clinicians should account for these variations by being cautious when interpreting the measurements from different devices, particularly in patients having cataract surgery with toric IOLs, where minimal differences in biometry measurements can alter postoperative refractive outcomes [11]. These variations may lead to over- or under-correction of astigmatism, misalignment of the toric IOL axis, and refractive surprises, especially in eyes with prior refractive surgery or irregular corneas (e.g., keratoconus) [25]. To minimise variability, we recommend using the same device for all measurements in a single patient to reduce device-specific errors, ensuring regular calibration, and performing multiple measurements when errors are suspected.

Traditional keratometry relies on a fixed refractive index (usually 1.3375), which assumes a linear relationship between anterior and posterior curvature. However, this assumption becomes unreliable in conditions such as prior refractive surgery, corneal ectasia, or irregular corneas, where the anterior to posterior curvature ratio is altered [26]. Consequently, the posterior cornea, which significantly contributes to total corneal power, is often overlooked. Studies have demonstrated that incorporating posterior corneal measurements into IOL calculations improves accuracy [11, 25] and helps avoid errors such as over- or under-correction in toric IOL implantation due to unaccounted posterior corneal astigmatism [11]. Additionally, total keratometry provides more accurate objective measure of corneal power in patient with refractive surgery compared to standard keratometry [27].

Our study is robust since it was designed prospectively, and a single investigator to scan both biometers. However, there are also limitations to consider. Due to the variability in the effect size (Cohens d = 0.07 to 1.2) of the biometer parameters, some effects may not have been detected due to insufficient power. Future studies should include a larger sample size to produce a large effect size for all variables and should be conducted as a multicentre study to account for the variability limited by a single-centre study. This will increase the generalisability due to a more diverse population. Our study assessed the agreement between the IOLMaster 700 and Galilei G6 using a single measurement for each eye. While repeatability and reproducibility were not directly evaluated, future studies could further explore these factors to strengthen the understanding of these devices’ performance, as studies have shown discrepancies, particularly in moderate to high corneal cylinder measurements with the Galilei G6 [28]. Most participants enrolled in the study either had normal eyes or cataracts. Since our results indicate discrepancies in corneal parameters, and studies report that the agreement of biometric measurements taken with different principles tends to decline in cohort with keratoconus or those who have undergone post-refractive surgery compared to the normal cohort. Future studies should assess whether the agreement between the Galilei G6 and IOLMaster 700 biometers declines in such cohorts [29]. Additionally, the sequence of testing was not randomized; however, given that these are non-contact biometers, outcomes are less likely to be affected by the order of the measurements.

## Conclusion

In conclusion, our study indicates that the IOLMaster 700 and Galilei G6 Lens Professional devices are not suitable for the interchangeability of anterior chamber depth (ACD), posterior corneal metrics, and total corneal metrics. Further studies are needed to validate these findings and explore their applicability in unique cohorts, such as those with conditions like keratoconus and high myopia, where these metrics may have greater clinical relevance.

## Data Availability

The datasets are available upon reasonable request from the corresponding author.

## Abbreviations

ACD: Anterior chamber depth
AXL: Axial length
CCT: Central corneal thickness
Cyl: Cylinder power
D: Dioptre
ICC: Intraclass correlation coefficients
IOL: Intraocular lens
J0: Corneal astigmatism vector along the 0° meridian
J45: Corneal astigmatism vector along the 45° meridian
K1: Front flat corneal keratometry
K2: Front steep corneal keratometry
LoA: Limits of agreement
LT: Lens thickness
MD: Mean difference
SD: Standard deviation
SS-OCT: Swept-source optical coherence tomography
WTW: White-to-white
